# Relationship between change in social evaluation learning and mood in early antidepressant treatment: A prospective cohort study in primary care

**DOI:** 10.1177/02698811221116928

**Published:** 2022-08-24

**Authors:** Catherine Hobbs, Milly Beck, Faye Denham, Laura Pettitt, Julian Faraway, Marcus R Munafò, Jie Sui, David Kessler, Katherine S Button

**Affiliations:** 1 Department of Psychology, University of Bath, Bath, UK; 2 Department of Mathematical Sciences, University of Bath, Bath, UK; 3 School of Psychological Science, University of Bristol, Bristol, UK; 4 MRC Integrative Epidemiology Unit, University of Bristol, Bristol, UK; 5 National Institute of Health Research Biomedical Research Centre, University Hospitals Bristol NHS Foundation Trust and the University of Bristol, Bristol, UK; 6 School of Psychology, University of Aberdeen, Aberdeen, UK; 7 Population Health Sciences, University of Bristol, Bristol, UK

**Keywords:** Depression, antidepressants, self-schema, social cognition, emotional processing

## Abstract

**Background:**

Antidepressants are proposed to work by increasing sensitivity to positive versus negative information. Increasing positive affective learning within social contexts may help remediate negative self-schema. We investigated the association between change in biased learning of social evaluations about the self and others, and mood during early antidepressant treatment.

**Method:**

Prospective cohort assessing patients recruited from primary care in South West England at four timepoints over the first 8 weeks of antidepressant treatment (*n* = 29). At each timepoint, participants completed self-report measures of depression (Beck Depression Inventory II (BDI-II) and Patient Health Questionnaire 9 (PHQ-9)), anxiety (Generalised Anxiety Disorder Questionnaire 7 (GAD-7)), and a computerised task measuring learning of social evaluations about the self, a friend and a stranger.

**Results:**

We did not find evidence that learning about the self was associated with a reduction in PHQ-9 (*b* = 0.08, 95% CI: −0.05, 0.20, *p* = 0.239) or BDI-II scores (*b* = 0.10, 95% CI: −0.18, 0.38, *p* = 0.469). We found some weak evidence that increased positive learning about the friend was associated with a reduction in BDI-II scores (*b* = 0.30, 95% CI: −0.02, 0.62, *p* = 0.069). However, exploratory analyses indicated stronger evidence that increased positive learning about the self (*b* = 0.18, 95% CI: 0.07, 0.28, *p* = 0.002) and a friend (*b* = 0.22, 95% CI: 0.10, 0.35, *p* = 0.001) was associated with reductions in anxiety.

**Conclusions:**

Change in social evaluation learning was associated with a reduction in anxiety but not depression. Antidepressants may treat anxiety symptoms by remediating negative affective biases towards socially threatening information directed towards the self and close others. However, our findings are based on exploratory analyses within a small sample without a control group and are therefore at risk of type 1 errors and order effects. Further research with larger samples is required.

## Introduction

Depression is associated with increased sensitivity to negative relative to positive information across cognitive domains (Dalgleish and Watts, 1990; Dalili et al., 2015; Roiser and Sahakian, 2013). According to the cognitive neuropsychological theory, antidepressants work by remediating these negative affective biases early in treatment, increasing sensitivity to positive relative to negative affective information. An improvement in mood is later produced when individuals have interacted with their social environment with remediated affective biases, allowing them to relearn associations from a more positive perspective (Godlewska and Harmer, 2021; Harmer et al., 2003; Roiser et al., 2012).

To date, research examining the effect of antidepressants on affective processing has been conducted primarily within laboratory settings using short-term administration of antidepressants (see Godlewska and Harmer, 2021 for a summary of current research). A small number of studies have been conducted in primary care, with mixed findings. Supportive of the cognitive neuropsychological theory, a prospective cohort study found that patients starting antidepressant treatment became more accurate at recognising positive facial emotions, which correlated with a later improvement in mood (Tranter et al., 2009). In addition, a machine learning algorithm based on change in facial emotion recognition and baseline depression severity predicted patients response to citalopram with 77% accuracy (Browning et al., 2019). However, tailoring treatment based on this algorithm was not beneficial in reducing depression (although benefits were found for anxiety) (Browning et al., 2021). Furthermore, a large-scale randomised controlled trial (RCT) in primary care patients found no effect of sertraline on recall of emotion words (Ahmed et al., 2021). At present, evidence for change in affective processing associated with antidepressant treatment in primary care settings is therefore inconsistent.

It may be possible to improve the current evidence base by identifying a more precise psychological mechanism. One such mechanism may be affective processing that maintains depressive self-schema. According to the cognitive model of depression, adverse social experiences in early life lead individuals to develop core sets of negative beliefs about the self, termed negative self-schemas. When negative self-schemas are activated by stressors in later life, they promote automatic processing of negative and punishing information about the self. Negative self-schema are therefore reinforced by affective biases in a vicious cycle (Beck, 1987; Beck and Dozois, 2011). Focusing on the role of antidepressants in remediating negative affective biases occurring in reference to self-schema may therefore provide a more sensitive measure of antidepressant action.

Social evaluation learning is believed to be a key mechanism linking change in affective processing to change in self-schema. Perceptions of the self are informed by how we believe others view us (Shrauger and Schoeneman, 1979). Within social interactions, healthy individuals demonstrate greater sensitivity to positive feedback (Button et al., 2015; Korn et al., 2012). Conversely, individuals experiencing depression preferentially engage with negative social evaluations (Giesler et al., 1996), and show poorer learning of positive social evaluations about the self (Hobbs et al., 2021). Repeated exposure to negative social feedback about the self is likely to reinforce negative self-schema. In line with the cognitive neuropsychological model, antidepressants may operate by increasing sensitivity to positive social feedback, remediating negative self-schema by exposing individuals to increased positive evaluations about the self.

We investigated whether antidepressants increase positive learning of social evaluations about the self and if change in social evaluation learning was associated with a change in depression. We took a naturalistic approach, observing change in social evaluation learning in primary care patients prescribed an antidepressant under the care of their general practitioner (GP) over the first 8 weeks of treatment. We hypothesised that patients would become better at learning positive social evaluations about the self and that this would be associated with a reduction in depression.

## Method

The study protocol was pre-registered on Open Science Framework (https://osf.io/z9p8a), where study materials are also available (https://osf.io/8a95j/). The data that support the findings of this study are openly available in the University of Bath Research Data Archive at https://doi.org/10.15125/BATH-01107 (Hobbs et al., 2022).

### Participants

Participants were recruited through GP referrals from primary care sites in South West England. Eligible patients were aged 18–65 years, fluent in English, with normal or corrected-to-normal vision. Patients were eligible if they were considering antidepressant treatment but had not yet started treatment or were within the first 2 weeks of antidepressant treatment. A wash-out period of 8 weeks was required between previous and current courses of antidepressants.

Exclusion criteria included experience of a mental health disorder or developmental difficulties other than depression and anxiety (e.g. bipolar disorder, psychosis, autism, personality disorder and/or eating disorders), current treatment for substance misuse and receiving care or being referred to secondary mental healthcare services. Due to the potential influence on affective processing, we excluded participants receiving cognitive behavioural therapy at baseline. We also excluded patients prescribed amitriptyline, pregabalin, benzodiazepines or any major tranquiliser during the study or 8 weeks prior to participating.

Data were collected at four main timepoints: baseline, 2-, 6- and 8-week follow-up. An additional long-term follow-up timepoint was completed at 6 months by a small proportion of participants. Prior to the COVID-19 pandemic, data were collected face to face by researchers. To account for social distancing measures, from April 2020 onwards, all data were collected remotely using online survey (Qualtrics, 2020) and cognitive task (Inquisit, 2020) software.

### Ethical approval

All participants provided written or digital informed consent. Ethical approval was obtained from the South West Frenchay NHS Research Ethics Committee (18/SW/0287).

### Measures

At each timepoint, participants completed self-report measures of mood and a computerised cognitive task measuring social evaluation learning.

#### Self-report measures of mood

We used the Patient Health Questionnaire 9 (PHQ-9) as a primary measure of depression, and the Beck Depression Inventory II (BDI-II) as a secondary measure. Both questionnaires measure depression symptoms in the preceding 2 weeks with greater scores indicating greater severity. We used the Generalised Anxiety Disorder Questionnaire (GAD-7) to measure generalised anxiety symptoms in the preceding 2 weeks.

We used a single-item global rating of change (GRC) scale (‘How have your moods and feelings changed?’) to measure participants’ perceptions of change in mood. At baseline, participants were asked to respond based on change in the previous 2 weeks, at follow-up participants were asked to respond based on change since the previous timepoint. Following previous research (Hobbs et al., 2020a), we collated GRC responses into a binary outcome of feeling better versus the feeling the same or worse to reflect that neither feeling the same nor worse is a positive therapeutic outcome.

At baseline only, participants completed the Clinical Interview Schedule-Revised (CIS-R) (Lewis et al., 1992), a self-administered computerised assessment that determines International Statistical Classification of Diseases and Related Health Problems 10th Revision (ICD-10) diagnoses of common mental health disorders. The CIS-R was completed in face-to-face sessions only.

#### Social evaluation learning task

**Figure 1.**
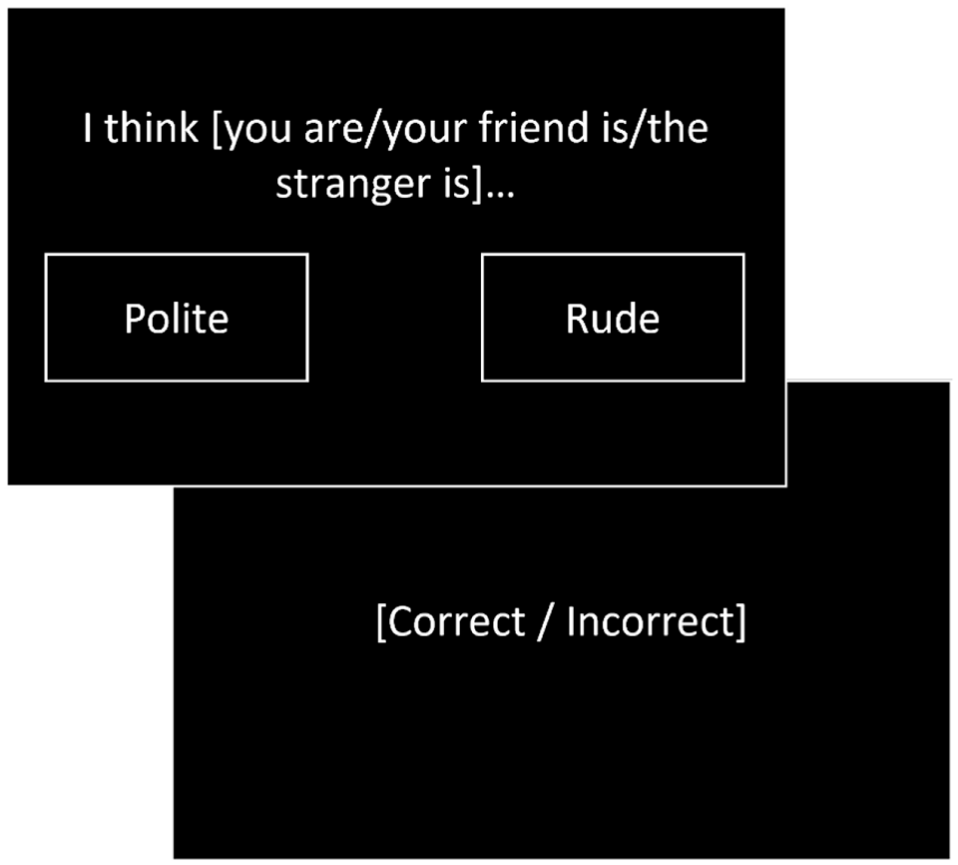
Example of a trial in the social evaluation learning task. Participants learnt how much the computer ‘liked’ or ‘disliked’ the self, a friend and a stranger in separate blocks based on feedback to selection of positive or negative social evaluation words. Participants learnt two rules, a positive ‘like’ rule where ‘correct’ feedback was given upon selection of the positive evaluation on 60%–80% of trials, and a negative ‘dislike’ rule where ‘correct’ feedback was given upon selection of positive evaluations on 20%–40% of trials.

We used a computerised social evaluation learning task to measure affective learning within social contexts (Button et al., 2015). Participants learnt how much the computer ‘liked’ the self, a friend and a stranger based on feedback to a forced choice selection between positive and negative social evaluation pairings (Figure 1). Participants learnt two rules based on the probability of the positive evaluations being ‘correct’ (positive ‘like’ 60%–80%, negative ‘dislike’ 20%–40%). No time limit was imposed on selection of words. Individual blocks were completed for each referential condition and rule. Order of referential condition, and rule nested within referential condition, was randomised. Participants completed 24 trials per referential condition–rule block.

To measure learning, we calculated the number of errors made before reaching the criterion of eight consecutive rule-congruent responses. We then calculated bias scores to reflect learning of the positive relative to the negative rule, by subtracting errors to criterion made when learning the negative rule from the positive rule. Lower scores indicate a more positive bias as more errors have been made learning the negative relative to the positive rule.

#### Additional measures

Additional measures were completed by participants prior to COVID-19. Full details are reported in Supplemental Materials. To allow for remote data collection and to reduce potential fatigue effects, only the measures outlined above were completed by participants following the pandemic. We chose to focus on the social evaluation learning task as we have previously found a reliable relationship between task outcomes and depression symptoms (Hobbs et al., 2019, 2021), as well as evidence of modulation by antidepressant administration (Hobbs et al., 2020b). Due to low statistical power associated with a reduced sample size, aside from the CIS-R which has been included for descriptive purposes, these data have not been analysed and is not reported within this paper. However, we have made all outcome data from these measures openly available.

### Statistical analyses

Analyses were conducted in R version 4.0.5.

#### Sample Size

We aimed to recruit 52 participants to provide 80% power to detect small to medium effects (*d_z_* = 0.40), equivalent to an approximately two-point change in social evaluation learning bias scores with an assumed standard deviation of 5. However, the COVID-19 pandemic occurred midway through the study impacting recruitment. Based on the number of participants that provided data at baseline and follow-up timepoints, we are powered to detect medium to large within-subject effects (*d_z_* = 0.61), equivalent to an approximately three-point change in bias scores.

*Hypothesis 1: Social evaluation learning regarding the self will become more positively biased during antidepressant treatment, as measured by a better learning of positive evaluations relative to negative evaluations towards the self.*

We used mixed-effects linear regression models with bias scores (positive errors to criterion − negative errors to criterion) as a continuous outcome, participant as a random effect and timepoint as categorical predictors. In addition, to investigate the specificity of effects to the self, we included referential condition and an interaction between referential condition and timepoint as categorical predictors.

*Hypothesis 2: Change in social evaluation learning will be associated with a reduction in depressive symptoms, as indicated by a decrease in PHQ-9 scores.*

We calculated change in PHQ-9 scores and bias scores in each referential condition separately by subtracting the current timepoint from the previous timepoint. We then used a mixed-effect linear regression model with change in PHQ-9 scores as a continuous outcome and change in bias scores in each referential condition as predictors. Timepoint was entered as a fixed effect and participant as a random effect to account for the repeated measures design. As change in depression is influenced by baseline severity (Bauer-Staeb et al., 2021), we entered baseline PHQ-9 scores as a fixed effect. To assess the reliability of these findings, we repeated this analysis with another measure of depression, the BDI-II.

### Exploratory analyses

We explored whether change in social evaluation learning was associated with a change in anxiety symptoms by repeating the analysis for hypothesis 2, substituting GAD-7 scores for PHQ-9 scores. To assess whether these findings persisted when co-morbid depression was taken into account, we repeated this analysis adjusting for depression by including change in PHQ-9 and BDI-II scores as additional predictors.

## Results

### Sample

**Figure 2.**
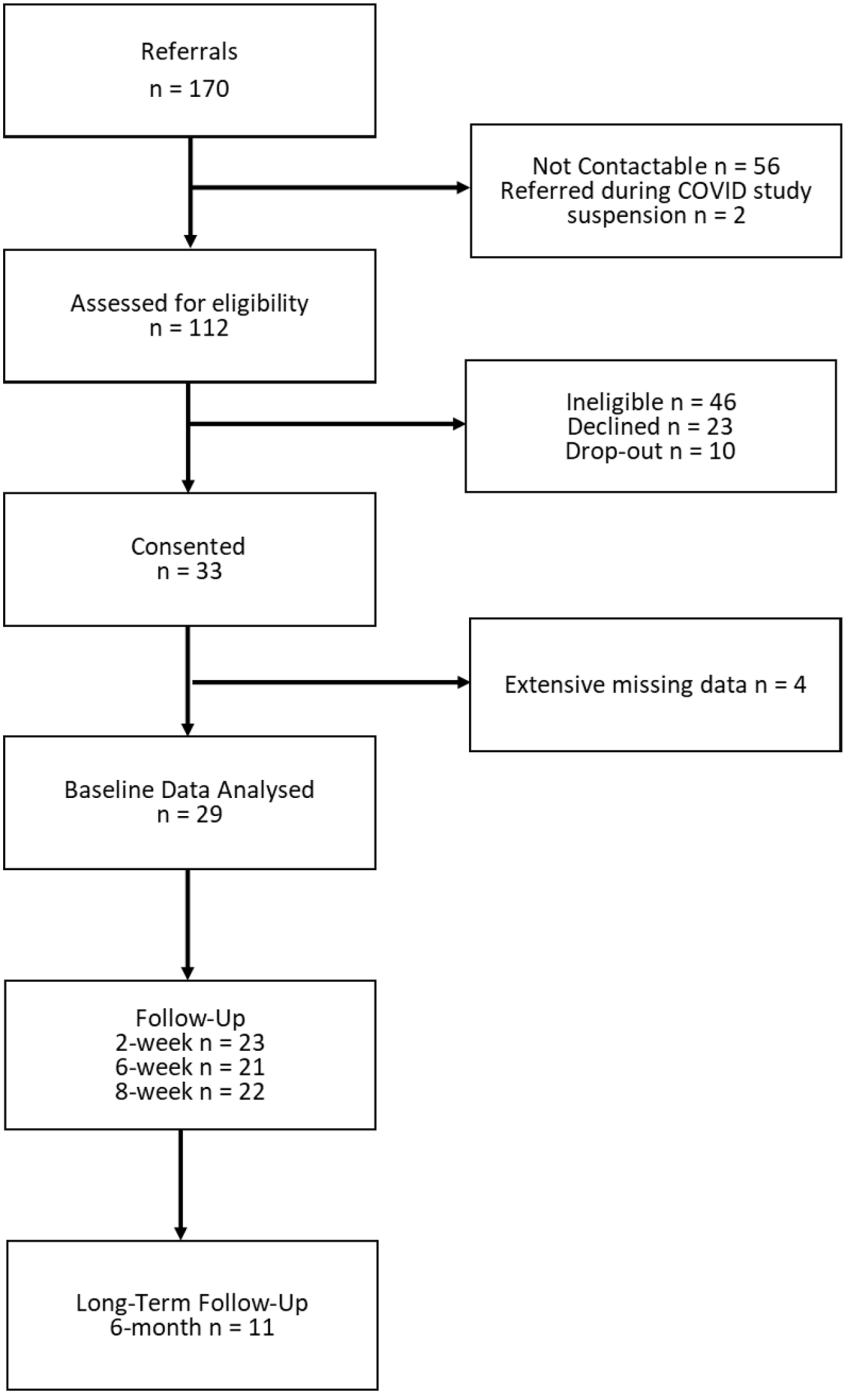
Flow diagram illustrating participant recruitment.

Of 170 patients referred to the study, 33 patients took part in the baseline data collection session (Figure 2). Four participants with baseline data only were excluded due to extensive missing data, leaving 29 participants for analysis. At 2-, 6- and 8-week follow-up, 23 (79%), 21 (72%) and 22 (76%) participants provided data, respectively. In all, 11 participants (38%) provided data at 6-month long-term follow-up.

Baseline sample characteristics are presented in Table 1. Patients were aged 18–60 years (mean: 38, standard deviation: 11), predominantly female (62%), and all identified as white. At baseline, all but one participant was taking an antidepressant for an average of 9 days. The most common antidepressants were sertraline (54%) and citalopram (32%). Approximately half of the patients reported previous antidepressant treatment.

**Table 1.** Baseline sample characteristics.

*N*	29
Age, *M* (SD)	38.3 (11.3)
Gender, *N* (%)	
Male	11 (37.9)
Female	18 (62.1)
Ethnicity, *N* (%)	
White	29 (100)
Occupation, *N* (%)	
Employed	22 (75.9)
Student	2 (6.9)
Unemployed	3 (10.3)
Other	2 (6.9)
Educational attainment^a^, *N* (%)	
GCSE or equivalent	5 (17.2)
A-Level or equivalent	8 (27.6)
Diploma or equivalent	6 (20.7)
Degree or equivalent	10 (34.5)
Relationship, *N* (%)	
Married/living as married	17 (58.6)
Single	8 (27.6)
Divorced/separated	4 (13.8)
Living situation, *N* (%)	
Homeowner	17 (58.6)
Renting	4 (13.8)
Living with a relative/friend	8 (27.6)
Depression duration, *N* (%)	
<2 weeks	0 (0)
2 weeks–6 months	9 (32.1)
6 months–1 year	6 (21.4)
1–2 years	2 (7.1)
⩾2 years	11 (39.3)
CIS-R primary diagnosis^b^	
Depressive episode	17 (89.5%)
Generalised anxiety disorder	2 (10.5%)
CIS-R secondary diagnosis^b^	
Generalised anxiety disorder	8 (42.1%)
Mixed anxiety and depression	8 (42.1%)
Specific phobia	3 (15.8%)
Antidepressant medication, *N* (%)^c^	
Sertraline	15 (53.6)
Citalopram	9 (32.1)
Fluoxetine	2 (7.1)
Mirtazapine	2 (7.1)
Length of current antidepressant treatment (days), *M* (SD)	9.3 (3.7)
Other medications^d^, *N* (%)	7 (24.1)
Previous antidepressant treatment, *N* (%)	14 (48.3)
Previous psychological therapy, *N* (%)	12 (41.4)
Data collection, *N* (%)	
Face to face	19 (65.5)
Remote	10 (34.5)

A-Level: Advanced Level; CIS-R: Clinical Interview Schedule Revised; GCSE: General Certificate of Secondary Education; SD: standard deviation.

a GCSEs are an entry-level qualification taken by UK students typically at the end of compulsory education at 16 years of age, equivalent to O-Levels or CSEs; A-Levels are a post-16 pre-university subject-specific qualification, equivalent to the International Baccalaureate; Diploma or equivalent are practical worked-based qualifications in specific industries/careers, includes Regulated Qualification Framework, National Vocation Qualification or Business and Technology Education Council diplomas; Degree or equivalent refers to undergraduate or postgraduate degree or higher qualification completed at a university or other higher education institution.

b The CIS-R was completed in face-to-face baseline testing sessions only. Data are therefore only available for 19 participants.

c One participant was not taking antidepressant medication at baseline.

d Medication reported included treatment for diabetes and high blood pressure, hormonal treatment, treatment for an underactive thyroid gland, an asthma inhaler, medication for heartburn, antibiotics, painkillers and the contraceptive pill.

Details of treatment characteristics by timepoint are reported in Supplemental Table S1. Two participants discontinued antidepressant treatment (*n* = 1, 2 weeks and *n* = 1, 6 weeks). There was high treatment adherence across timepoints. A small proportion of participants reported also receiving psychological therapy (7%–19% across timepoints).

### Change in mood

Mean scores for measures of depression (PHQ-9 and BDI-II) and anxiety (GAD-7) are reported in Table 2. At baseline, participants on average experienced moderate depression and anxiety symptoms. Both depression and anxiety declined over time; by 8-week follow-up average scores reflected mild symptoms. Most participants reported feeling better at follow-up timepoints on the GRC.

**Table 2.** Descriptive statistics for self-report measures of depression (PHQ-9 and BDI-II), anxiety (GAD-7), change in mood (GRC) and bias scores in the social evaluation learning task by timepoint. Greater bias scores indicate a more negative bias (relatively better learning of the negative relative to the positive rule).

	Baseline	2 weeks	6 weeks	8 weeks	6 months
Self-reported mood					
*N*	29	23	21	21	11
PHQ–9, *M* (SD)	14.31 (4.81)	9.09 (5.01)	7.62 (4.84)	6.90 (5.39)	8.73 (6.39)
BDI–II, *M* (SD)	26.50 (8.74)	19.78 (9.71)	13.53 (8.55)	13.62 (10.70)	12.10 (9.17)
GAD–7, *M* (SD)	12.83 (4.72)	8.26 (4.74)	6.38 (5.45)	5.29 (4.74)	7.45 (6.12)
GRC, *N* (%)^a^					
Worse/same	15 (53.57)	4 (17.39)	6 (28.57)	3 (14.29)	1 (9.09)
Better	13 (46.43)	19 (82.61)	15 (71.43)	18 (85.71)	10 (90.91)
Social evaluation learning bias scores					
* N*	29	22	20	21	10
Self, *M* (SD)	−0.84 (8.30)	−2.34 (7.22)	−2.85 (7.67)	−2.48 (5.69)	−6.05 (7.77)
Friend, *M* (SD)	−5.28 (8.18)	−4.23 (7.20)	−4.00 (6.57)	−4.07 (6.59)	−2.94 (8.57)
Stranger, *M* (SD)	−1.36 (5.60)	−3.16 (6.05)	−2.33 (6.16)	−1.45 (6.71)	−7.56 (8.58)

BDI-II scores were missing for one participant at baseline, two participants at 6 weeks and one participant at 6 months. Bias scores were missing for the friend and stranger conditions for one participant at 6-month follow-up.

BDI-II: Beck Depression Inventory II; bias scores: positive errors to criterion − negative errors to criterion; GAD-7: Generalised Anxiety Disorder Questionnaire; GRC: Global Rating of Change Scale; PHQ-9: Patient Health Questionnaire 9; SD: standard deviation.

a At baseline, participants responded to the question ‘Compared to 2 weeks ago, how have your moods and feelings changed?’, at follow-up timepoints participants responded to the question ‘Compared to when you last answered these questions, how have your moods and feelings changed?’

*Hypothesis 1: Social evaluation learning regarding the self will become more positively biased during antidepressant treatment, as measured by a better learning of positive evaluations relative to negative evaluations towards the self.*

**Figure 3.**
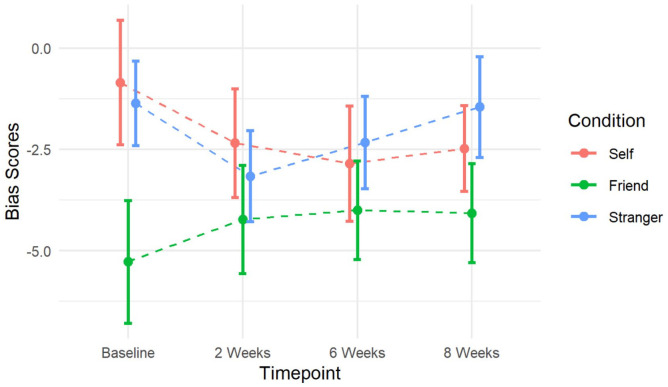
Change in bias scores according to referential condition over 8 weeks of antidepressant treatment. Lower bias scores indicate a more positive bias as participants have made a greater number of errors learning the negative ‘dislike’ rule versus the positive ‘like’ rule. Error bars represent standard errors.

Participants were more positively biased when learning about the friend versus the self at baseline (*b* = −4.43, 95% CI: −7.76, −1.11, *p* = 0.010). However, there was no evidence of a change in bias scores over time, or that this differed by referential condition (Table 3; session: *p* = 0.934, session × referential condition: *p* = 0.834). We therefore did not find evidence to support our hypothesis. Full results are reported in Table 3 and mean bias scores are presented in Table 2 and Figure 3.

**Table 3.** Mixed-effects linear regression models examining differences in SEL bias scores (outcome) by timepoint and referential condition.

	*b*	*b*, 95% CI	β	β, 95% CI	*p*
Intercept	−0.85	−3.38, 1.69	0.29	−0.08, 0.65	0.514
Session					0.934
Baseline	Reference				
2 weeks	−1.57	−5.18, 2.03	−0.23	−0.75, 0.29	0.393
6 weeks	−2.26	−5.97, 1.45	−0.33	−0.86, 0.21	0.234
8 weeks	−1.83	−5.48, 1.83	−0.26	−0.79, 0.26	0.328
Referential condition					0.160
Self	Reference				
Friend	−4.43	−7.76, −1.11	−0.64	−1.12, −0.16	0.010
Stranger	−0.52	−3.84, 2.81	−0.07	−0.55, 0.40	0.761
Session × referential condition					0.834
2 weeks × friend	2.55	−2.52, 7.61	0.37	−0.36, 1.10	0.326
6 weeks × friend	3.28	−1.92, 8.49	0.47	−0.28, 1.22	0.218
8 weeks × friend	2.84	−2.29, 7.97	0.41	−0.33, 1.15	0.280
2 weeks × stranger	−0.30	−5.36, 4.76	−0.04	−0.77, 0.69	0.907
6 weeks × stranger	1.04	−4.16, 6.25	0.15	−0.60, 0.90	0.695
8 weeks × stranger	1.54	−3.59, 6.67	0.22	−0.52, 0.96	0.557

*b*: unstandardised regression coefficients, β: standardised regression coefficient; SEL: social evaluation learning.

*Hypothesis 2: Change in social evaluation learning will be associated with a reduction in depressive symptoms, as indicated by a decrease in PHQ-9 scores.*

We did not find evidence in support of our hypothesis. Change in depression severity, measured by PHQ-9 scores, was not associated with change in biased learning about the self (*b* = 0.08, 95% CI: −0.05, 0.20, *p* = 0.239), the friend (*b* = 0.09, 95% CI: −0.05, 0.23, *p* = 0.229) or the stranger (*b* = −0.01, 95% CI: −0.15, 0.13, *p* = 0.871; Table 4, Supplemental Figure 1).

**Table 4.** Mixed-effects linear regression models examining the association between change in PHQ-9/BDI-II/GAD-7 scores (outcomes) and change in SEL bias scores by referential condition.

	*b*	*b*, 95% CI	β	β, 95% CI	*p*
PHQ-9					
Intercept	−2.02	−5.58, 1.55	−0.52	−0.90, −0.14	0.272
Bias scores change					
Self	0.08	−0.05, 0.20	0.15	−0.10, 0.39	0.239
Friend	0.09	−0.05, 0.23	0.15	−0.09, 0.38	0.229
Stranger	−0.01	−0.15, 0.13	−0.02	−0.27, 0.23	0.871
Baseline PHQ-9	−0.22	−0.44, −0.01	−0.24	−0.47, −0.01	0.050
Session					0.003
Baseline to 2 weeks	Reference				
2–6 weeks	3.97	1.41, 6.53	0.86	0.31, 1.42	0.004
6–8 weeks	3.62	1.03, 6.22	0.79	0.22, 1.35	0.008
BDI-II					
Intercept	1.52	−6.32, 9.37	−0.25	−0.65, 0.15	0.705
Bias scores change					
Self	0.10	−0.18, 0.38	0.10	−0.17, 0.36	0.469
Friend	0.30	−0.02, 0.62	0.24	−0.01, 0.49	0.069
Stranger	−0.03	−0.37, 0.31	−0.02	−0.29, 0.25	0.874
Baseline BDI-II	−0.32	−0.58, −0.07	−0.31	−0.56, −0.07	0.016
Session					0.039
Baseline to 2 weeks	Reference				
2–6 weeks	0.90	−4.64, 6.45	0.10	−0.50, 0.70	0.751
6–8 weeks	6.37	0.89, 11.85	0.69	0.10, 1.29	0.027
GAD-7					
Intercept	−0.17	−3.38, 3.03	−0.34	−0.66, −0.02	0.917
Bias Scores Change					
Self	0.18	0.07, 0.28	0.34	0.14, 0.55	0.002
Friend	0.22	0.10, 0.35	0.36	0.16, 0.56	0.001
Stranger	0.01	−0.11, 0.13	0.02	−0.19, 0.23	0.835
Baseline GAD-7	−0.33	−0.55, −0.11	−0.30	−0.50, −0.10	0.004
Session					0.020
Baseline to 2 weeks	Reference				
2–6 weeks	2.98	0.79, 5.16	0.65	0.17, 1.12	0.010
6–8 weeks	1.97	−0.25, 4.19	0.43	−0.05, 0.91	0.088

*b* = unstandardised regression coefficients, β = standardised regression coefficient; BDI-II: Beck Depression Inventory II; GAD-7: Generalised Anxiety Disorder Questionnaire; PHQ-9: Patient Health Questionnaire 9; SEL: social evaluation learning.

When we examined the association between change in learning and BDI-II scores, our secondary measure of depression, we found weak evidence of an association in the friend condition. An increase in learning of positive relative to negative evaluations about the friend was associated with a reduction in BDI-II scores, although statistical evidence for this was weak (*b* = 0.30, 95% CI: −0.02, 0.62, *p* = 0.069). We did not find evidence of an association with biased learning about the self or the stranger (Table 4, Supplemental Figure 1).

### Exploratory analysis

In exploratory analyses, we found evidence of an association between change in anxiety and change in bias scores (Table 4, Supplemental Figure 1). Increased positive learning about the self and the friend, indicated by a reduction in bias scores, was associated with a small reduction in GAD-7 scores (Self: *b* = 0.18, 95% CI: 0.07, 0.28, *p* = 0.002, friend: *b* = 0.22, 95% CI: 0.10, 0.35, *p* = 0.001). We did not find evidence of an association between change in anxiety and learning about a stranger (*b* = 0.01, 95% CI: −0.11, 0.13, *p* = 0.835). When we adjusted for change in PHQ-9 and BDI-II scores, these findings were unchanged (Supplemental Table S2).

### Long-term follow-up

At 6-month follow-up participants on average showed a small increase in PHQ-9 and GAD-7 scores from 8-week follow-up, although scores remained substantially lower than baseline. Bias scores declined for the self and the stranger but remained relatively stable for the friend (Table 2). However, as only a small proportion of participants provided data at this timepoint (*n* = 11, 37.9%), the majority of whom reported feeling better (90.91%), this pattern may only represent a particular subset of participants.

## Discussion

We investigated the association between change in learning of social evaluations and depression symptoms over the first 8 weeks of antidepressant treatment in primary care patients. We hypothesised that antidepressants may remediate negative self-schema by increasing learning of positive social evaluations about the self. However, we did not find evidence of an association between change in learning about the self and a reduction in PHQ-9 or BDI-II scores. Despite most patients showing an improvement in depression, learning about the self was relatively stable. These findings are in line with our recently published research, where acute citalopram did not influence learning about the self in healthy volunteers (Hobbs et al., 2020b). It is possible that self-schemas may be more effectively targeted using a combination of antidepressants and cognitive behavioural therapy to address both top-down and bottom-up affective biases (Dozois et al., 2009; Roiser et al., 2012). However, our findings do not support the theory that change in self-referential learning of social evaluations plays a central role in addressing depression symptoms in antidepressant treatment alone.

In contrast to our hypotheses, which focused on the role of social evaluation learning in depression, exploratory analyses indicated that change in affective learning was associated with a reduction in anxiety. On average, patients that became better at learning positive versus negative evaluations about both the self and the friend showed a reduction in anxiety symptoms. Cognitive models propose that individuals with generalised anxiety hold self-schemas focused on personal threat which lead to biased processing of threatening environmental information (Beck, 1976). We have previously found that individuals with greater social anxiety show better learning of negative relative to positive social evaluations (Button et al., 2012, 2015). Negative social evaluations, such as criticism, may represent a source of social threat that individuals with generalised anxiety implicitly interpret as potential acts of social exclusion or aggression. Antidepressants may reduce sensitivity to negative information, helping to remediate these threat-related biases and subsequently reducing anxiety symptoms.

Our findings are similar to those of a recent RCT within primary care. Patients experiencing depression whose treatment was guided by an algorithm based on change in facial emotion recognition show a greater decline in the secondary outcome of anxiety, but not depression (Browning et al., 2021). Previous research has also found that short-term antidepressant treatment of generalised anxiety reduced threat-related interpretative biases (Mogg et al., 2004). Furthermore, recent evidence suggests that change in anxiety rather than depression may be a more sensitive measure of therapeutic outcome of antidepressants (Lewis et al., 2019). Researchers have subsequently proposed that focusing on change in affective processing as a predictor of anxiety may be more useful than depression (Browning et al., 2021). Our findings suggest that change in social evaluation learning about the self and familiar others may be a sensitive marker of antidepressant response based on change in anxiety symptoms.

In contrast to our hypotheses, which focused on learning about the self, we found weak evidence of an association between increased positive learning about a friend and a reduction in BDI-II scores. Although these findings are based on weak evidence using our secondary outcome of depression, they are in line with our exploratory analyses which also found an association between learning about the friend and a reduction in anxiety. In addition, in our recently published research we found that an acute dose of citalopram in healthy volunteers increased learning of positive characteristics in friends but not the self (Hobbs et al., 2020b). Substantial evidence suggests that antidepressants are associated with increased positive social behaviours (Young et al., 2014). It is possible that antidepressants operate in part by enhancing sensitivity to positive characteristics in familiar others, leading to greater prosocial behaviours. This may increase engagement in social interactions, addressing issues of social withdrawal and anhedonia associated with depression.

### Strengths

Whereas most previous research examining affective processing in antidepressant action has been conducted under controlled laboratory settings, we took a naturalistic approach observing primary care patients receiving antidepressant treatment. Our findings are therefore more representative of antidepressant treatment for depression and anxiety in the United Kingdom. We also employed a prospective cohort design, allowing us to investigate the relationship between change in mood and social evaluation learning occurring over time.

In addition, we employed a novel task that was able to integrate self, emotion and reward processing. We have previously validated the use of this task in individuals experiencing varying levels of depression (Hobbs et al., 2019, 2021) and anxiety (Button et al., 2012, 2015; Hopkins et al., 2021).

### Limitations

Recruitment was impacted by the COVID-19 pandemic, and we were unable to reach our target sample. Due to our small sample, we were underpowered to detect small effects heightening the likelihood of type 1 and 2 errors (Button et al., 2013; Vadillo et al., 2016). In addition, our sample was limited in its demographic diversity with all participants identifying as white. Although antidepressant response has not been found to differ according to ethnicity (Lesser et al., 2010), our findings may not be reflective of all individuals within primary care. Further research evaluating our findings in a larger and more demographically diverse sample is required.

This study lacked a control group limiting our ability to understand the causal role of change in social evaluation learning on anxiety or depression and increasing risk of order effects. It is possible that change in social evaluation learning may be an epiphenomenon of mood disorders rather than playing a mechanistic role. Recruiting larger samples would allow for more complex statistical analyses, such as cross-lagged regression models (Hecht and Zitzmann, 2021), to investigate this possibility.

## Conclusions

In contrast to our hypotheses, we did not find evidence of an association between change in social evaluation learning and depression symptoms. Change in social evaluation learning was instead more reliably associated with a reduction in anxiety. Patients that became more positively biased when learning about both the self and the friend on average showed a reduction in anxiety symptoms. Antidepressants may treat anxiety symptoms by remediating negative affective biases towards socially threatening information. However, these findings are based on exploratory analyses and require further replication.

## Supplemental Material

sj-docx-1-jop-10.1177_02698811221116928 – Supplemental material for Relationship between change in social evaluation learning and mood in early antidepressant treatment: A prospective cohort study in primary care Supplemental material, sj-docx-1-jop-10.1177_02698811221116928 for Relationship between change in social evaluation learning and mood in early antidepressant treatment: A prospective cohort study in primary care by Catherine Hobbs, Milly Beck, Faye Denham, Laura Pettitt, Julian Faraway, Marcus R Munafò, Jie Sui, David Kessler and Katherine S Button in Journal of Psychopharmacology
