# Supplementary material for: Relationship between change in social evaluation learning and mood in early antidepressant treatment: A prospective cohort study in primary care
Source: J Psychopharmacol. 2022 Aug 24;37(3):303–12. doi: 10.1177/02698811221116928 (PMC10076340; doi:10.1177/02698811221116928)
Supplement: sj-docx-1-jop-10.1177_02698811221116928 – Supplemental material for Relationship between change in social evaluation learning and mood in early antidepressant treatment: A prospective cohort study in primary care [file sj-docx-1-jop-10.1177_02698811221116928.docx]

## Supplementary Materials

Hobbs, C., Beck, M., Denham, F., Pettit, L., Faraway, J., Munafò, M.R., Sui, J., Kessler, D., & Button, K. S. (2020). The relationship between change in social evaluation learning and mood in early antidepressant treatment: a prospective cohort study in primary care.

## Additional measures collected prior to the COVID-19 pandemic

Additional questionnaire measures and cognitive tasks were completed by participants prior to the COVID-19 pandemic. However, to allow for remote data collection and to reduce potential fatigue effects, we removed the measures outlined below.

### Associative Learning

We used three tasks to examine different aspects of associative learning (self, reward and valence) occurring independently (Hobbs et al., 2021; Stolte et al., 2016; Sui and Humphreys, 2015). Participants completed the tasks sequentially, however the order was counterbalanced across time-points and participants. At the beginning of each task, participants were instructed to remember specified stimuli-abstract shape pairings. Stimuli consisted of the labels ‘self’, ‘friend’ and ‘stranger’ for the self task, the labels ‘£9’, ‘£3’ and ‘£1’ for the reward task, and a happy, neutral and sad cartoon face for the valence task. In each trial a fixation point was initially presented for 200 ms, followed by a stimuli-shape pairing for 100-150 ms. Participants used the keyboard to indicate whether the presented pairings matched with the pairings specified at the beginning of the task. Two blocks of 60 trials were completed per task. Accuracy and reaction times were recorded.

### Facial Emotion Recognition

Participants completed a facial emotion recognition task at each time-point, measuring accuracy in recognising six emotions (happiness, sadness, fear, anger, disgust, surprise) (Griffiths et al., 2015). In each trial a fixation cross appeared in the centre of the screen for 1500-2500 ms. This was then replaced by a face stimulus for 250ms, followed by a mask of visual noise for 250ms. Text labels for the six possible emotions then appeared on the screen in a circle formation. Participants were asked to select the emotion that they thought was displayed. This was a forced choice response although no time limit was imposed. Participants completed one block of 96 trials. Accuracy and reaction times were recorded.

### Word Categorisation and Recall

Participants categorised whether 40 presented positive and negative traits described themselves or a familiar other (Hobbs et al., 2020). Traits were presented for 500 ms, and participants used the keyboard to indicate their response (yes/no). Participants were then given two minutes to recall these traits, using the keyboard to enter their responses. Separate blocks were completed for each referential condition in a randomised order. Recalled traits were recorded.

### Self-Report Measures of Mood

At each timepoint participants completed the Brief Fear of Negative Evaluation Scale (Leary, 1983) as a measure of social anxiety, the Rosenberg Self-Esteem Scale (Rosenberg, 1965) as a measure of self-esteem, and the 24 item version of the Dysfunctional Attitudes Scale (Power et al., 1994) as a measure of maladaptive self-schema. To measure change in state affect during testing participants completed the positive and negative affect scale (PANAS) (Watson et al., 1988) before and after completion of the cognitive tasks at each timepoint.

Supplementary Table S1

*Treatment Characteristics by Timepoint*

|  | **Baseline** | **2-weeks** | **6-weeks** | **8-weeks** | **6-months** |
| --- | --- | --- | --- | --- | --- |
| **N** | 29 | 23 | 21 | 22 | 11 |
| **Current Antidepressant, N (%)** | 28 (96.6) | 23 (100.0) | 19 (95.0) | 20 (95.2) | 8 (72.7) |
| **Antidepressant Medication, N (%)** |  |  |  |  |  |
| **Sertraline** | 15 (53.6) | 11 (47.8) | 9 (47.4) | 11 (55.0) | 7 (87.5) |
| **Citalopram** | 9 (32.1) | 9 (39.1) | 8 (42.1) | 8 (40.0) | 1 (12.5) |
| **Fluoxetine** | 2 (7.1) | 2 (8.7) | 2 (10.5) | 0 (0) | 0 (0) |
| **Mirtazapine** | 2 (7.1) | 0 (0.0) | 0 (0.0) | 0 (0) | 0 (0) |
| **Mirtazapine and sertraline** | 0 (0) | 1 (4.4) | 0 (0) | 1 (5.0) | 0 (0) |
| **Change in Antidepressant Treatment ^a^, N (%)** |  |  |  |  |  |
| **Discontinuation** | - | 1 (4.4) | 1 (5.0) | 0 (0) | 3 (27.3) |
| **Medication ^b^** | - | 2 (9.1) | 0 (0) | 0 (0) | 0 (0) |
| **Dose** ^c^ | - | 1 (4.8) | 2 (11.1) | 7 (36.8) | 3 (37.5) |
| **Adherence ^d^** |  |  |  |  |  |
| **Taken tablets everyday** | 27 (96.4) | 18 (78.3) | 12 (63.2) | 14 (70.0) | 6 (75.0) |
| **Taken nearly all of tablets** | 1 (3.6) | 5 (21.7) | 7 (36.8) | 6 (30.0) | 2 (25.0) |
| **Side Effects, N (%)** | 14 (50.0) | 11 (47.8) | 6 (31.6) | 4 (20.0) | 2 (25.0) |
| **Current Psychological Therapy, N (%)** | 2 (6.9) | 2 (8.7) | 2 (9.5) | 4 (19.1) | 0 (0) |
| **Other Medication, N (%)** | 7 (24.1) | 7 (30.4) | 5 (23.8) | 5 (23.8) | 2 (18.2) |
| **Data Collection, N (%)** |  |  |  |  |  |
| **Face to face** | 19 (65.5) | 15 (65.2) | 13 (61.9) | 15 (71.4) | 4 (36.4) |
| **Remote** | 10 (34.5) | 8 (34.8) | 8 (38.1) | 6 (28.6) | 7 (63.6) |

^a^ Change from previous timepoint

^b^ One participant had treatment augmented with an additional antidepressant (mirtazapine to mirtazapine & sertraline), one participant switched from sertraline to fluoxetine

^c^ Aside from one participant at 8-week follow-up who had a reduction in dose, all changes in doses of medication were an increase.

^d^ Possible options to the statement ‘As regards your antidepressants, I have...’ were (1) ‘Taken my tablets everyday’ (2) ‘Taken nearly all of my tablets’, (3) ‘Taken more than half of my tablets’, (4) ‘Taken less than half of my tablets’, (5) ‘Taken hardly any of my tablets’, (6) ‘Not taken any of my tablets’. No participants selected options 3-6.

Note: Percentages reflect proportions of participants within timepoints. For antidepressant medication, change in antidepressant treatment, adherence, and reported side effects only participants that reported currently taking an antidepressant responded, percentages therefore reflect the proportions of patients currently receiving an antidepressant within timepoints.

Supplementary Table S2

*Mixed-effect linear regression models examining the association between change in biased learning of social evaluations (predictors) and change in GAD-7 scores (outcome) adjusting for change in PHQ-9 and BDI-II scores*

|  | ***b*** | ***b* 95% CI** | **β** | **β 95% CI** | **p** |
| --- | --- | --- | --- | --- | --- |
| **Adjusting for change in PHQ-9 scores** | | | | | |
| Intercept | 0.49 | -1.94, 2.91 | -0.04 | -0.31, 0.23 | 0.697 |
| Bias Scores Change |  |  |  |  |  |
| Self | 0.13 | 0.04, 0.21 | 0.25 | 0.08, 0.41 | 0.004 |
| Friend | 0.17 | 0.08, 0.27 | 0.28 | 0.13, 0.44 | 0.001 |
| Stranger | 0.02 | -0.07, 0.11 | 0.04 | -0.12, 0.21 | 0.600 |
| Baseline GAD-7 | -0.15 | -0.32, 0.02 | -0.14 | -0.30, 0.02 | 0.095 |
| Session |  |  |  |  | 0.630 |
| Baseline to 2-weeks | Reference |  |  |  |  |
| 2 to 6-weeks | 0.65 | -1.15, 2.44 | 0.14 | -0.26, 0.54 | 0.485 |
| 6 to 8-weeks | -0.07 | -1.86, 1.72 | -0.02 | -0.41, 0.38 | 0.936 |
| PHQ-9 Change | 0.58 | 0.40, 0.75 | 0.57 | 0.39, 0.76 | < .001 |
| **Adjusting for change in BDI-II scores** | | | | | |
| Intercept | 0.37 | -2.13, 2.87 | -0.17 | -0.44, 0.09 | 0.773 |
| Bias Scores Change |  |  |  |  |  |
| Self | 0.12 | 0.03, 0.21 | 0.23 | 0.06, 0.41 | 0.011 |
| Friend | 0.19 | 0.09, 0.30 | 0.30 | 0.14, 0.46 | 0.001 |
| Stranger | 0.08 | -0.03, 0.19 | 0.13 | -0.05, 0.31 | 0.163 |
| Baseline GAD-7 | -0.20 | -0.38, -0.03 | -0.18 | -0.34, -0.02 | 0.029 |
| Session |  |  |  |  | 0.003 |
| Baseline to 2-weeks | Reference |  |  |  |  |
| 2 to 6-weeks | 2.62 | 0.88, 4.35 | 0.56 | 0.18, 0.94 | 0.005 |
| 6 to 8-weeks | -0.02 | -1.85, 1.81 | 0.00 | -0.40, 0.40 | 0.984 |
| BDI-II Change | 0.27 | 0.18, 0.35 | 0.52 | 0.35, 0.69 | < .001 |

*b* = unstandardised regression coefficients, β = standardised regression coefficient.


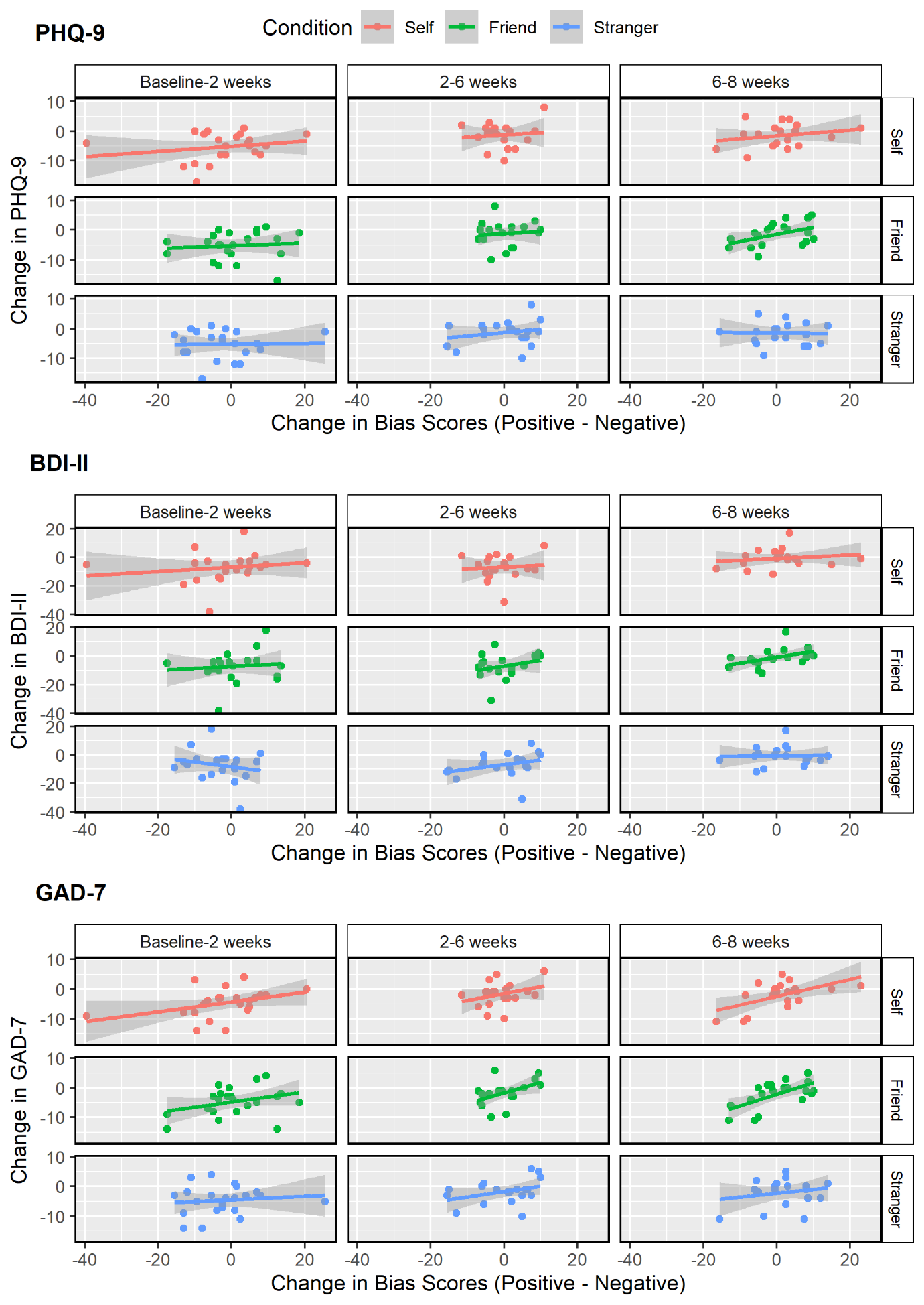


*Supplementary Figure 1*

Association between change in biased learning about the self, a friend and a stranger and self-report measures depression (PHQ-9 and BDI-II) and anxiety (GAD-7) by timepoint. Errors bars represent 95% confidence intervals.

## References

Griffiths S, Penton-Voak IS, Jarrold C, et al. (2015) No own-age advantage in children’s recognition of emotion on prototypical faces of different ages. *PLoS ONE* 10(5): e0125256. DOI: 10.1371/journal.pone.0125256.

Hobbs C, Murphy SE, Wright L, et al. (2020) Effect of acute citalopram on self-referential emotional processing and social cognition in healthy volunteers. *BJPsych Open* 6(6). DOI: 10.1192/bjo.2020.107.

Hobbs C, Sui J, Kessler D, et al. (2021) Self-processing in relation to emotion and reward processing in depression. *Psychological Medicine*: 1–13. DOI: 10.1017/s0033291721003597.

Leary MR (1983) A Brief Version of the Fear of Negative Evaluation Scale. *Personality and Social Psychology Bulletin* 9(3). Sage PublicationsSage CA: Thousand Oaks, CA: 371–375. DOI: 10.1177/0146167283093007.

Power MJ, Katz R, McGuffin P, et al. (1994) The Dysfunctional Attitude Scale (DAS). A Comparison of Forms A and B and Proposals for a New Subscaled Version. *Journal of Research in Personality* 28(3). Academic Press: 263–276. DOI: 10.1006/jrpe.1994.1019.

Rosenberg M (1965) Rosenberg self esteem scle. *Personality and Individual Differences*. DOI: 10.1007/s12671-015-0407-6.

Stolte M, Humphreys G, Yankouskaya A, et al. (2016) Dissociating biases towards the self and positive emotion. *Quarterly Journal of Experimental Psychology* 70(6): 1011–1022. DOI: 10.1080/17470218.2015.1101477.

Sui J and Humphreys GW (2015) The interaction between self-bias and reward: Evidence for common and distinct processes. *Quarterly Journal of Experimental Psychology* 68(10): 1952–1964. DOI: 10.1080/17470218.2015.1023207.

Watson D, Clark LA and Tellegen A (1988) Development and validation of brief measures of positive and negative affect: The PANAS scales. *Journal of Personality and Social Psychology* 54(6): 1063. DOI: 10.1037//0022-3514.54.6.1063.
